# Ruptured subscapular artery aneurysm and subclavian artery occlusion in a patient with type 1 neurofibromatosis: a case report

**DOI:** 10.1186/1752-1947-8-39

**Published:** 2014-02-05

**Authors:** Antoine Moreau, Julien Joskin, Julie Kreutz, Alain Nchimi

**Affiliations:** 1 Medical student, Liège University, Department of Medical Imaging, University Hospital Sart Tilman, Domaine du Sart Tilman B35, 4000, Liège, Belgium; 2 Department of Medical Imaging, University Hospital –Sart Tilman, Domaine du Sart Tilman B35, 4000 Liège, Belgium

**Keywords:** Aneurysm embolization, Neurofibromatosis, Subscapular artery

## Abstract

**Introduction:**

Collateral muscular artery aneurysm is exceedingly rare. We report the first case of subscapular artery aneurysm in a patient with type 1 neurofibromatosis and ipsilateral chronic subclavian artery occlusion.

**Case presentation:**

A 74-year-old Caucasian woman with a medical history of type 1 neurofibromatosis, presented a sudden left pectoral mass, later diagnosed as a ruptured aneurysm of the left subscapular artery. It was caused by a chronic occlusion of the left subclavian artery, diagnosed on angiographies prior to embolization.

**Conclusions:**

Collateral artery aneurysm in the event of a mainstream muscular artery chronic occlusion may occur in type 1 neurofibromatosis.

## Introduction

Type 1 neurofibromatosis (NF1) is an autosomal dominant disorder that affects one out of 3000 individuals and mainly involves neuroectodermal tumors in the peripheral nervous system [1]. Vascular abnormalities may occur in 0.4 to 6.4% of patients [2]. The spectrum of vascular disease includes stenosis, obstruction and aneurysms. On the other hand, arterial chronic total occlusion may cause collateral artery aneurysm. This finding is exceptional in muscular arteries. Here we report the case of a patient with NF1 who underwent a subscapular aneurysm rupture as the consequence of a subclavian artery chronic total occlusion.

## Case presentation

**Figure 1:**
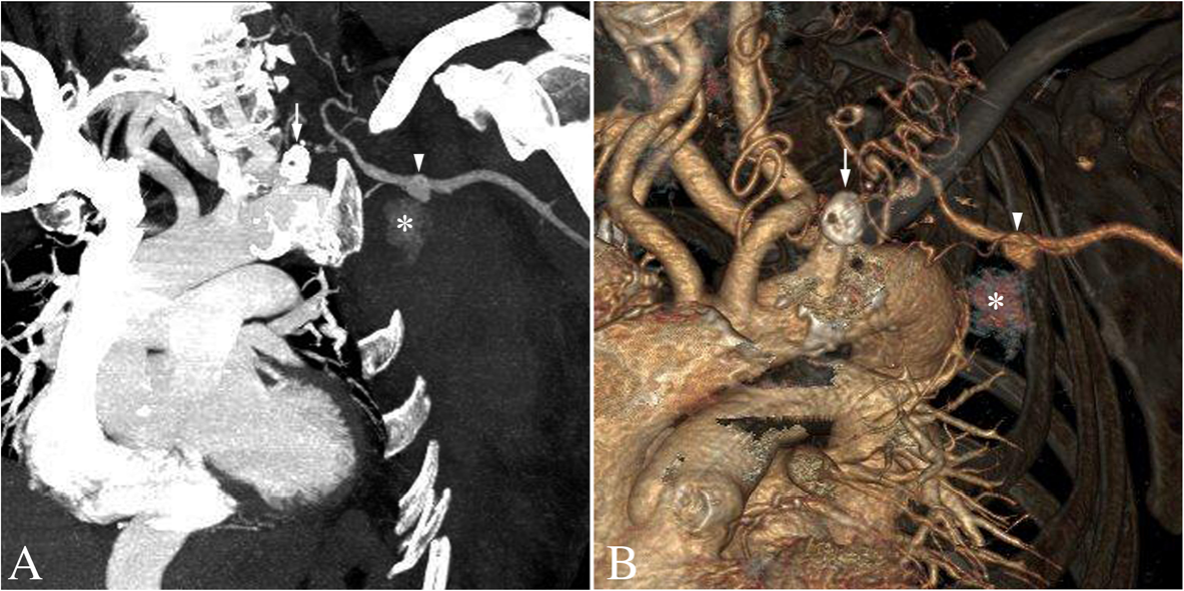
**Contrast-enhanced computed tomography of aneurysm of the left subscapular artery.** Oblique maximum intensity **(A)** and volume rendering **(B)** projections of contrast-enhanced computed tomography show a 1cm-diameter aneurysm of the left subscapular artery (arrowheads), associated with a blush of contrast agent (asterisks). More proximally, there is an extensively calcified chronic total occlusion of the proximal left subclavian artery (arrows). Of note, there is an aberrant origin of the right subclavian artery.

A 74-year-old Caucasian woman was admitted to our emergency department for a pulsatile left pectoral pain and axillary mass that had suddenly appeared three hours before. She denied any history of trauma, thoracic outlet syndrome or central vein cannulation. She had a medical history of hypertension, hypercholesterolemia and NF1 with multiple resections of neurofibromas. A physical examination revealed multiple cutaneous neurofibromas and an axillary pulsatile, stiff and painful mass without erythema. All peripheral pulses were perceived. Blood pressure on her right arm was 135/65mmHg. The rest of the physical examination was unremarkable. Her hematocrit and hemoglobin levels were normal. A contrast-enhanced computed tomography (CT) scan showed a 1cm-large aneurysm on the root of the subscapular artery with signs of rupture consisting of a nodular blush of contrast surrounded by a large hematoma on its lower portion (Figure 1). CT also revealed a chronic proximal left subclavian artery occlusion, associated with an important collateral network depending on the left thyrocervical trunk branches and perfusing the left axillary artery through anastomoses with the left subscapular artery where the blood flow was retrograde on Doppler ultrasonography (not shown).

**Figure 2:**
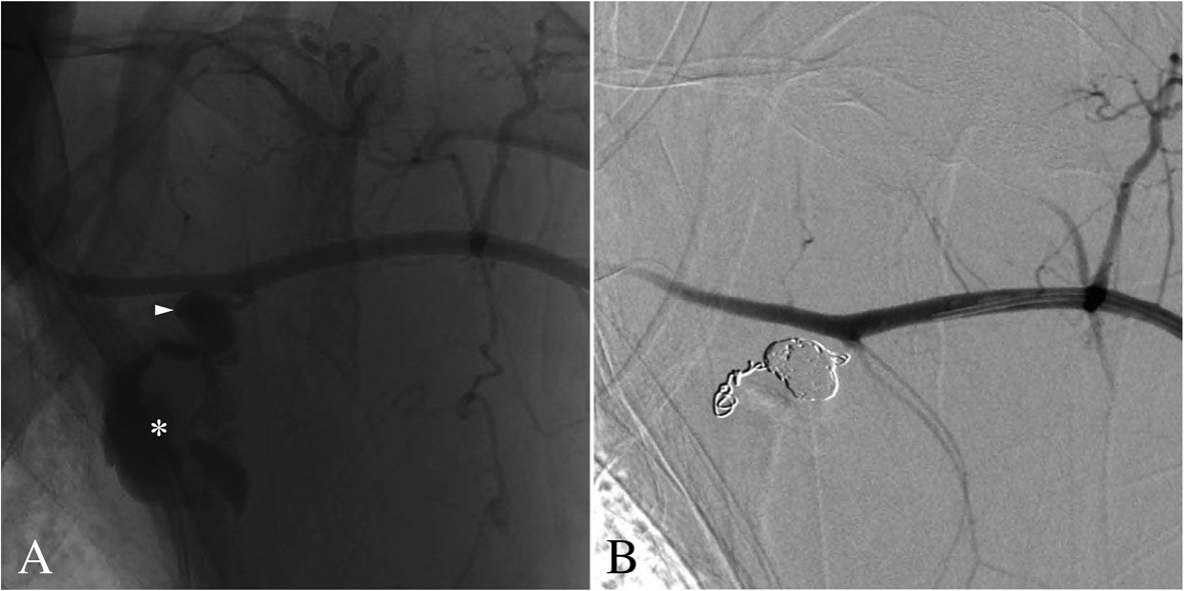
**Arteriography of aneurysm of the left subscapular artery. (A)** Catheter angiography shows an aneurysm on the proximal left subscapular artery (arrowhead), the bottom part of the aneurysm is ruptured into a larger pseudoaneurysmal collection (asterisk). **(B)** Angiography showed no residual bleeding after proximal and distal embolization of the subscapular artery and packing of the aneurysm.

Endovascular embolization was requested to control bleeding. An introducer sheath (Terumo Europe, Leuven, Belgium) was placed into the left brachial artery. Then, catheterization of the proximal part of the left subclavian artery with a 5 French vertebral catheter (Cook Europe, Bjaeverskov, Denmark) was performed. Subsequent angiography showed the ruptured aneurysm of the left subscapular artery (Figure 2A). Hyperselective catheterism of the afferent branch of this aneurysm was performed with a 2.7 French microcatheter (Progreat™, Terumo Europe, Leuven, Belgium). It was embolized with 0.018 Inch microcoils (Tornado™ Embolization Microcoils, Cook Europe, Bjaeverskov, Denmark). Afterward, we packed the aneurysm and finally embolized the efferent artery with the same coils. Postembolization angiography revealed complete occlusion of the aneurysm. The axillary artery and its different branches were permeable and remained supplied by the collateral network (Figure 2B). Clinical evolution was excellent and our patient was discharged without further event on day four postembolization.

## Discussion

In the above-reported case, a ruptured aneurysm on the left subscapular artery was successfully treated by endovascular embolization in a patient with NF1 and unsuspected chronic occlusion of the left subclavian artery. In such cases, a collateral network develops to maintain sufficient distal blood flow. As a result, the persistent antegrade or retrograde collateral overflow in small collateral arteries cause excess pressure that may lead to weakening of the arterial wall and occurrence of a true aneurysm. These abnormalities are well known from collateral aneurysms resulting from celiac trunk occlusion [3,4]. However, to the best of our knowledge, very few aneurysms resulting from occlusion of a mainly muscular artery have been reported; most in the upper arms [5]. This may be due to a greater hypoxic autoregulation, putting a higher pressure on collateral visceral arteries as compared to the muscular arteries.

We could not be certain that the subclavian artery occlusion was caused by NF1. However, no other sign of atherosclerosis was evident elsewhere, despite a history of hypertension and hypercholesterolemia (both medically controlled). Further, our patient denied any history of trauma, thoracic outlet syndrome or venous cannulation. We therefore speculate that atherosclerosis is less likely than NF1 to explain this previously unreported association of arterial abnormalities. Indeed, NF1 causes intimal proliferation, thinning of the muscle layer and fragmentation of the elastic layer [6] that may have promoted the vascular occlusion and later on, the aneurysm occurrence.

What is known about the management and prognosis of artery overflow aneurysms result from a series of pancreaticoduodenal aneurysms and could be summarized as follows: first, CT is probably the best noninvasive option for the diagnosis of these aneurysms, especially in ruptured cases. In addition, CT allows easy diagnosis of associated chronic total occlusion and provides important information for treatment planning. In selected cases, Doppler ultrasound may be useful to depict the flow pattern within involved vessels.

Second, endovascular treatment, surgery or both in combination have been described. Surgery has a higher operative and postoperative complication rate and is more difficult when the aneurysm has ruptured [7]. An endovascular approach is therefore the best therapeutic alternative, since it can be combined with revascularization of the causative occlusion or stenosis. The procedure depends on the need to preserve the aneurysmal artery flow. When the artery must stay patent, endovascular treatment options include covered stentgrafting, flow diversion, coil or liquid embolization of the aneurysmal sac. In other cases, an option is to completely embolize the artery by coiling both its upstream and downstream portions. In some cases, only antegrade blood flow restoration from the upstream occlusion stenting changes the flow dynamics in the aneurysm and may lead to a progressive thrombosis of the aneurysmal sac and eventually auto-occlusion. These different endovascular options can be considered on their own or combined [7].

Third, long-term outcomes are excellent for aneurysms treated by endovascular approach and neither bleeding nor recurrence of the aneurysm has been reported after successful embolization. Since, in our case, the causal gene defect and subclavian artery occlusion remain, a theoretical risk of developing another collateral aneurysm should be considered.

Last, it is unknown whether incidentally discovered aneurysms should be treated or not. The risk of rupture appears to be unrelated to the age of the patient and the size of the aneurysm [6,7]. However growing aneurysms can be considered more likely to rupture than stable aneurysms [8].

## Conclusions

We report a ruptured left subscapular artery collateral overflow aneurysm caused by subclavian left artery chronic occlusion, successfully treated by endovascular coil embolization. This previously unreported association may have been promoted by the known vascular frailty associated to NF1.

## Consent

Written informed consent for the publication of this case report and any accompanying images was obtained from the patient. A copy of the written consent is available for review by the Editor-in-Chief of this journal.

## Abbreviations

CT: computed tomography; NF1: type 1 neurofibromatosis.

## Competing interests

The authors declare that they have no competing interests.

## Authors’ contributions

JJ and JK were involved in the diagnosis, findings, interpretation and management of the case. AM, JJ and AN wrote the manuscript. All authors read and approved the final manuscript.
